# Long-term natural history data in Duchenne muscular dystrophy ambulant patients with mutations amenable to skip exons 44, 45, 51 and 53

**DOI:** 10.1371/journal.pone.0218683

**Published:** 2019-06-25

**Authors:** Claudia Brogna, Giorgia Coratti, Marika Pane, Valeria Ricotti, Sonia Messina, Adele D’Amico, Claudio Bruno, Gianluca Vita, Angela Berardinelli, Elena Mazzone, Francesca Magri, Federica Ricci, Tiziana Mongini, Roberta Battini, Luca Bello, Elena Pegoraro, Giovanni Baranello, Stefano C. Previtali, Luisa Politano, Giacomo P. Comi, Valeria A. Sansone, Alice Donati, Enrico Bertini, Francesco Muntoni, Nathalie Goemans, Eugenio Mercuri, on behalf on the International DMD group

**Affiliations:** 1 Pediatric Neurology, Department of Woman and Child Health and Public Health, Child Health Area, Università Cattolica del Sacro Cuore, Rome, Italy; 2 Centro Clinico Nemo, Fondazione Policlinico Universitario Agostino Gemelli IRCCS, Rome, Italy; 3 Dubowitz Neuromuscular Centre, UCL & Great Ormond Street Hospital, London, United Kingdom; 4 NIHR Great Ormond Street Hospital Biomedical Research Centre, London, United Kingdom; 5 Department of Clinical and Experimental Medicine, University of Messina, Messina, Italy; 6 Nemo SUD Clinical Centre, University Hospital “G. Martino”, Messina, Italy; 7 Department of Neurosciences, Unit of Neuromuscular and Neurodegenerative Disorders, Bambino Gesù Children’s Hospital, Rome, Italy; 8 Center of Myology and Neurodegenerative Disorders, Istituto Giannina Gaslini, Genoa, Italy; 9 Child Neurology and Psychiatry Unit, ‘‘Casimiro Mondino” Foundation, Pavia, Italy; 10 Fondazione IRCCS Ca’ Granda Ospedale Maggiore Policlinico, Dino Ferrari Centre, Department of Pathophysiology and Transplantation, University of Milan, Milan, Italy; 11 Neuromuscular Center, AOU Città della Salute e della Scienza, University of Torino, Torino, Italy; 12 Department of Developmental Neuroscience, Stella Maris Institute, Pisa, Italy; 13 Department of Clinical and Experimental Medicine, University of Pisa, Pisa, Italy; 14 Department of Neurosciences, University of Padua, Padua, Italy; 15 Fondazione IRCCS Istituto Neurologico Carlo Besta, Milan, Italy; 16 Division of Neuroscience, IRCCS San Raffaele Scientific Institute, Milan, Italy; 17 Dipartimento di Medicina Sperimentale, Seconda Università di Napoli, Napoli, Italy; 18 The NEMO Center in Milan, Neurorehabilitation Unit, University of Milan, ASST Niguarda Hospital, Milan, Italy; 19 Metabolic Unit, A. Meyer Children's Hospital, Florence, Italy; 20 Department of Child Neurology, University Hospitals Leuven, Leuven, Belgium; University of Rome La Sapienza, ITALY

## Abstract

**Introduction:**

The aim of this international collaborative effort was to report 36-month longitudinal changes using the 6MWT in ambulant patients affected by Duchenne muscular dystrophy amenable to skip exons 44, 45, 51 or 53.

**Materials and methods:**

Of the 92 patients included in the study, 24 had deletions amenable to skip exon 44, 27 exon 45, 18 exon 51, and 28 exon 53. Five patients with a single deletion of exon 52 were counted in both subgroups skipping exon 51 and 53.

**Results:**

The difference between subgroups amenable to skip different exons was not significant at 12 months but became significant at both 24 (p≤0.05) and 36 months (p≤0.01).

**Discussion:**

Mutations amenable to skip exon 53 had lower baseline values and more negative changes than the other subgroups while those amenable to skip exon 44 had better results both at baseline and at follow up. Deletions amenable to skip exon 45 were associated with a more variable pattern of progression. Single exon deletions were more often associated with less drastic changes but this was not always true in individual cases.

**Conclusion:**

Our results confirm that the progression of disease can differ between patients with different deletions, although the changes only become significant from 24 months onwards. This information is relevant because there are current clinical trials specifically targeting patients with these subgroups of mutations.

## Introduction

The introduction of glucocorticoid therapy and the implementation of standards of care have, in the last decades, dramatically changed survival and natural history of patients affected by Duchenne muscular dystrophy (DMD)[1, 2]. More recently, there has been increasing evidence that an additional benefit may come from new therapeutically approaches based on genetic mechanisms targeting specific types of mutation [3, 4]. Recent clinical trials using dystrophin restoration approaches, such as antisense oligonucleotides for exons skipping or approaches targeting non-sense mutations have shown encouraging results. The results appear to be more obvious after the first year of treatment [3]. The interpretation of the long-term results of the clinical trials however is complicated by the paucity of long-term prospective natural history data in patients with distinct genotypes.

Retrospective data on loss of ambulation and other features of disease progression [5, 6] in patients with deletions amenable to skip exon 44, 45, 51 and 53 have recently become available, but only few have reported prospectively collected longitudinal data in these subgroups of patients[7–9]. These papers suggest that the changes in the individual subgroups do not appear to be significantly different from the mean changes observed in the whole cohort of DMD patients over a year [7], but the difference becomes more obvious on a longer follow up (24 months), especially when individual subgroups were compared to each other [8].

As there are a number of ongoing or planned studies targeting deletions amenable to skip exon 44, 45, 51 and 53 (NCT03218995, NCT03375255, NCT02500381, NCT02310906, NCT03508947, NCT02081625), it has become important to obtain more detailed information on the patterns of progression related to these different genotypes.

The aim of our collaborative effort was to obtain longitudinal prospective changes over 36 months in the six minutes walking test (6MWT) in a large cohort of DMD ambulant patients amenable to skip exons 44, 45, 51 and 53.

## Materials and methods

The study is part of a longitudinal multi-centric cohort study involving 12 tertiary neuromuscular centers in Italy, 1 center in Belgium and one in the United Kingdom. Patients were recruited between January 2008 and March 2015 and followed for at least three years. Inclusion criteria at baseline were: genetically confirmed DMD diagnosis, patient still ambulant and able to walk independently for at least 150 meters, no severe or moderate intellectual disability or behavioral problems. In this paper, we only included patients with deletions amenable to skip exons 44, 45, 51 and 53. The study was approved by the Ethical Committee of each center (Catholic University, Rome, Ospedale Bambino Gesù, Rome; Istituto Mondino, Pavia; Gaslini Institute, Genova; Besta Institute, Milan; Stella Maris Institute, Pisa; University of Napoli, University of Messina; University of Torino; University of Padova; University of Milano; Centro Clinico Nemo Milano, University Hospitals Leuven, NHS Multicentric Research Ethics Committee, England). Parents of participants (all our patients were minor/children) and patients were informed that the data collected as part of our routine clinical assessment were going to be used anonymously for an observational study defining natural history of the diseases and they all gave written consent. Type and regime of steroids were also noted.

### 6MWT

All ambulant DMD boys performed 6MWT according to the ATS guidelines modified by having two examiners, one recording time and distances and one staying close to the patient for safety issues [10, 11]. As part of the routine assessments in all centers, patients are seen at least once every 12 months. Data were collected from the first assessment after recruitment (baseline) and at 12, 24, 36-months after baseline. Details of the training for the physiotherapists involved in the study and of the inter-observer reliability for 6MWT among the centers have already been reported [12, 13].

### Time to rise from the floor (TRF)

As part of this study all boys performed at each visit the timed items including time to rise from the floor (TRF). The TRF was performed recording the time taken to complete the task. It measures the time taken to rise from supine to standing[14]. The number of DMD boys unable to perform the task at each time point was noted. The boys who were able to perform the task were subdivided into two groups, based on the ability to perform the task in less or more than 6 seconds. Data were collected from the first assessment after recruitment (baseline) and from the 12, 24, 36-month assessments.

### Statistical analysis

The 6MWT was evaluated longitudinally at 12, 24 and 36 months. Descriptive statistics (N, mean, SD, range) were used.

Analysis of variance model (ANOVA) was used to assess heterogeneity among groups of the six minutes walking distance (6MWD) at baseline and at 12, 24, 36-month assessments. The test was performed according to the type of skipping exons 44, 45, 51, 53 and the use of steroids at baseline (naive vs alternate or continuous).

Non-parametric Wilcoxon signed-rank test was used in the whole study group to compare differences in 12, 24, 36 months assessment adjusting for baseline 6MWT value (<350 mt vs ≥350 mt) and TRF (< or > 6 sec). P value was set at < 0.05.

## Results

Of the 354 DMD patients included in the overall DMD longitudinal study, 228 had deletions, with 131 having deletions amenable to skip exons 44, 45, 51 and 53.

Of these 131 DMD patients, 92 (mean age 7.86 years (SD±2.32) fulfilled the inclusion criteria and entered the study. The remaining 39 could not be recruited because lost at follow up (n = 8), or participating in clinical trials (n = 22) or because they had been followed for less than three years or had incomplete data (n = 9).

Twenty-four of the 92 had deletions amenable to skip for exon 44, 27 amenable to skip for exon 45, 18 had deletions amenable to skip for 51, and 28 had deletions amenable to skip exon 53. Five patients with a single deletion of exon 52 were counted in both subgroups skipping exon 51 and 53. Of the 92 boys, 35 were under 7 years of age at the time of the first assessment (12 not on steroids, 8 on alternate and 15 on continuous steroids), 57 were above 7 years (4 not on steroids, 34 on alternate and 19 on continuous steroids). None of the patients discontinued steroid treatment during the study.

### 6MWT

Table 1 reports details of the 6MWT in the whole group with deletions and in our study cohort with 92 patients amenable to skip exons 44, 45, 51 and 53. Twenty-one patients lost ambulation within 36 months from baseline. When the boys lost the ability to perform the 6MWT they were scored as 0 meters. The mean age at baseline of the boys who lost ambulation was 9.15 years (SD±1.71). Thirty-five of the 92 patients were younger than 7 years of age, 57 were 7 year or older. On the 6MWT a difference was found between the two groups under and above 7 years of age at 24 month (p = <0.05) and at 36-month assessment (p = < 0.05).

**Table 1:** Details of the 6MWT in the whole group and in the study cohort.

Group	N	Age at baseline, mean (SD)	6MWT at baseline, mean (SD)	Age at 24 months, mean (SD)	6MWT at 24 months, mean (SD)	Age at 36 months, mean (SD)	6MWT at 36 months, mean (SD)
**All deletions**	169	7.77 (±2.50)	375.51 (±77.76)	9.50 (±2.58)	341.28 (±146.96)	10.49 (±2.58)	293.21 (±183.31)
**All deletions amenable to skip 44, 45, 51 and 53**	92	7.86 (±2.32)	381.13 (±83.64)	9.55 (±2.39)	337.65 (±156.70)	10.53 (±2.41)	285.50 (±189.42)
**Amenable to exon skipping <7 years**	35	5.38 (±0.84)	379.68 (±56.39)	7.35 (±0.86)	441.80 (±71.96)	8.30 (±0.92)	388.01 (±125.55)
**Amenable to exon skipping** **≥7 years**	57	9.39 (±1.45)	382.03 (±97.13)	11.39 (±1.52)	321.94 (±156.29)	12.40 (±1.51)	222.56 (±195.29)

Thirty-four of the 92 patients had 6MWD < 350 mt, 58 had 6MWD ≥ 350 mt at baseline.

When we analyzed the 6MWT mean changes from baseline a difference was found between the two groups below and above 350 mt at 12 months (p≤0.001), 24 months (p≤0.001) and at 36 months (p≤0.001).

No difference on the 6MWT mean values was found among the different steroids groups (no steroids vs alternate vs continuous regime) at baseline, 12, 24 and 36 months.

### TRF

Fifty-six of the 92 patients had TRF below 6 sec and 31 had TRF above or equal to 6 seconds at baseline. No data were available from 5 patients (1 amenable to skip for exon 51, 2 exon 44 and 2 exons 45). Table 2 reports details of the TRF in the study cohort according to mutations amenable to skip exons 44, 45, 51 and 53.

**Table 2:** Baseline 12, 24, 36 months TRF according to mutations amenable to skip exons 44, 45, 51 and 53.

	Age	TRF (mean)				Unable to perform TRF (n)		
	Subgroups by skipping	Baseline	12 months	24 months	36 months	12 months	24 months	36 months
**Study cohort** **(n:92)**	**44 (n:22)**	4,49	5,73	6,02	8,68	1	2	3
	**45 (n:25)**	5,72	5,60	5,99	5,78	2	4	6
	**51 (n:17)**	7,36	5,46	7,41	5,32	3	5	7
	**53 (n:28)**	6,21	9,62	11,25	9,90	3	8	13

On the 6MWT a difference was found between the groups below and above 6 seconds on the TRF at baseline (*p*≤0.0001), 12 months (*p*≤0.0001), 24 months (*p*≤0.0001) at 36 months (*p*≤0.0001).

### Skip 44

**Fig 1:**
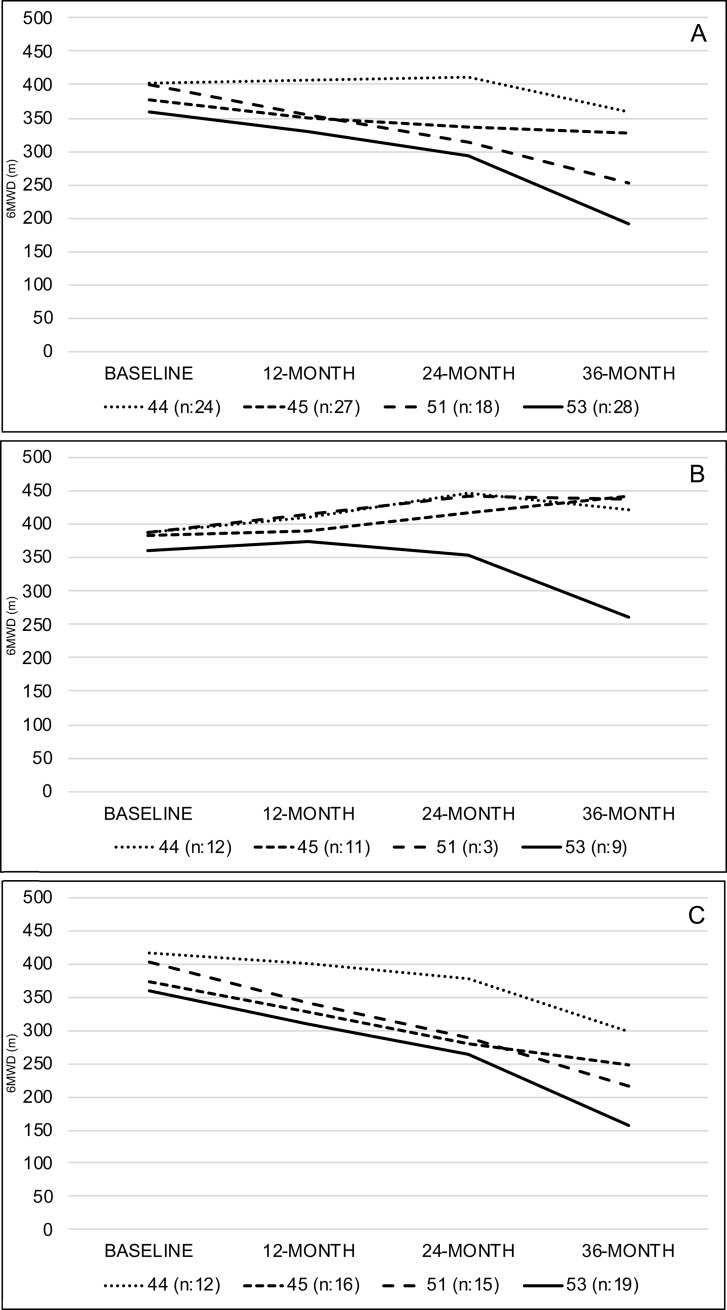
Mean 6MWT changes in the whole study cohort and in individual subgroups skipping exons 44, 45, 51 and 53 according to age. Panel A: Study Cohort. Panel B: <7 years of age. Panel C: ≥7 years of age.

**Fig 2:**
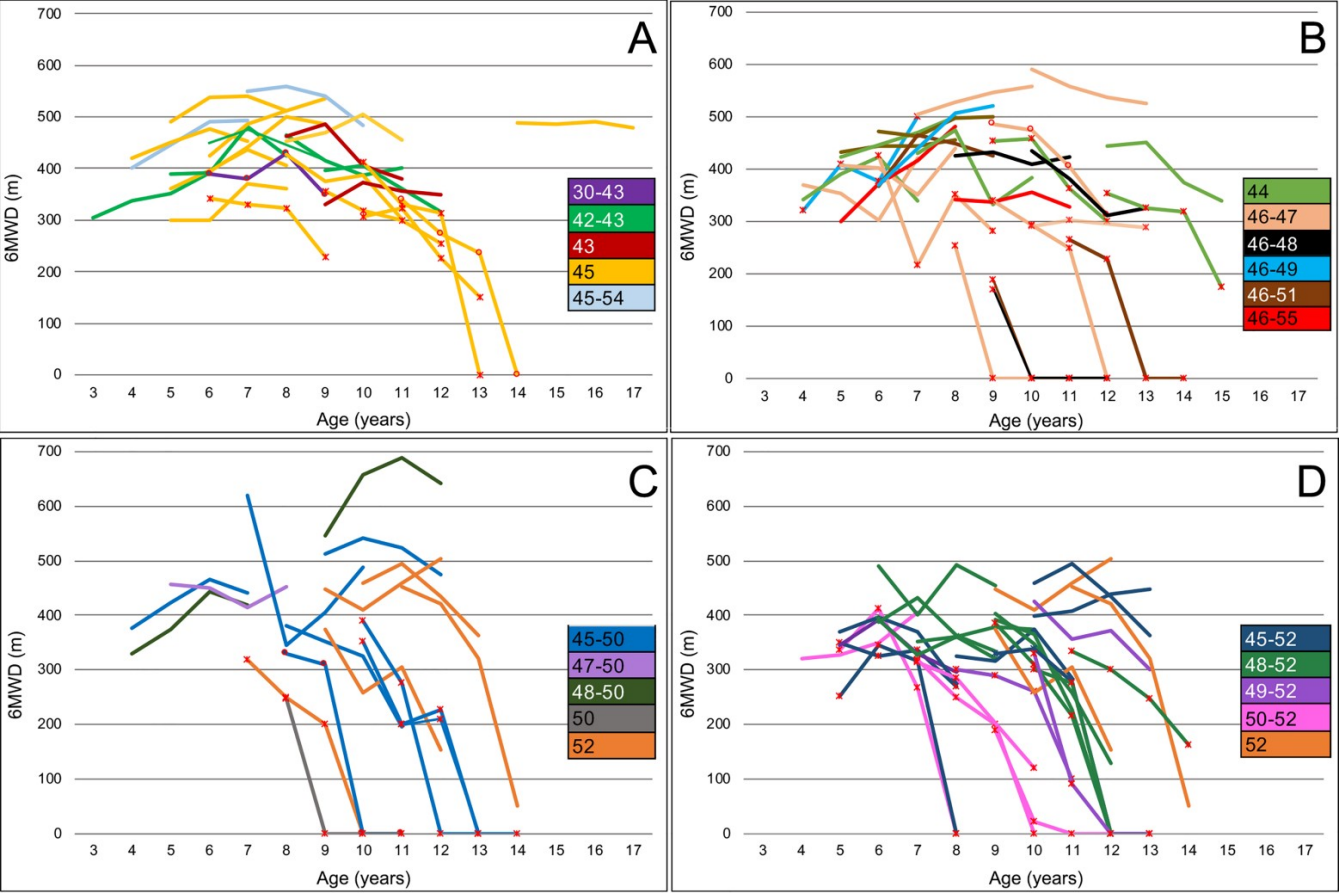
Individual 6MWT changes with details of the mutations within skipping subgroups. Panel A: Cohort skipping exon 44. Panel B: Cohort skipping exon 45. Panel C: Cohort skipping exon 51. Panel D: Cohort skipping exon 53. ✳ = TRF ≥ 6 sec, ○ = TRF not performed.

Twenty-four patients were amenable to skip exon 44. The mean 6MWT was of 402.25 mt at baseline. Mean changes from baseline were of +3.85 mt at 12 month +9.75 mt at 24 months and– 43.04 mt at 36 months. Twelve were younger than 7 years of age and 12 were above 7 years. (Table 3; Figs 1 and 2) Two lost ambulation at 36 months from baseline, their mean age at baseline was 10.5 years (SD ±0.71).

**Table 3:** Baseline 12, 24, 36 month 6MWT values (range, mean and median) subdivided according to genotype and age.

	AGE					6MWT						
	Subgroups by skipping	MIN	MEAN	MEDIAN	MAX	Baseline	12 months	24 months	36 months	12 month changes	24 month changes	36 month changes
**Study cohort** **(n:97)**	**44 (n:24)**	3.32	7.48	7.23	14.30	402.25	406.09	412.00	359.21	3.85	9.75	-43.04
	**45 (n:27)**	4.36	7.86	8.01	12.12	377.27	349.99	337.48	326.42	-27.28	-39.79	-50.85
	**51 (n:18)**	4.00	8.38	8.80	11.53	400.46	353.56	313.72	253.11	-46.90	-86.73	-147.34
	**53 (n:28)**	4.12	8.14	8.55	11.60	359.46	330.49	294.31	190.93	-28.97	-65.15	-168.54
**<7 years (n:33)**	**44 (n:12)**	3.32	5.45	5.78	6.90	388.66	411.28	446.82	420.67	22.62	58.16	32.01
	**45 (n:9)**	4.36	5.56	5.55	6.83	384.13	390.05	416.30	442.13	5.92	32.18	58.00
	**51 (n:3)**	4.00	4.56	4.08	5.60	386.83	415.67	441.67	437.33	28.83	54.83	50.50
	**53 (n:9)**	4.12	5.33	5.20	6.07	359.89	373.01	353.56	261.89	13.12	-6.33	-98.00
**≥** **7 years (n:64)**	**44 (n:12)**	7.57	9.52	9.10	14.30	415.83	401.33	377.18	297.75	-14.50	-38.65	-118.08
	**45 (n:18)**	7.00	9.45	9.33	12.12	372.56	327.46	279.68	246.88	-45.09	-92.88	-125.68
	**51 (n:15)**	7.00	9.15	9.14	11.53	403.18	341.13	288.13	216.27	-62.05	-115	-187
	**53 (n:19)**	7.00	9.47	9.76	11.60	359.26	310.35	264.69	157.32	-48.91	-95	-202

### Skip 45

Twenty-seven patients were amenable to skip exon 45. The mean 6MWT was of 377.27 mt at baseline. Mean changes from baseline were of -27.28 mt at 12 month, -39.79 mt at 24 months and -50.85 mt at 36 months. Eleven were younger than 7 years of age and 16 were above 7 years. (Table 3; Figs 1 and 2). Five lost ambulation at 36 months from baseline, their mean age at baseline was 9.59 years (SD ±0.86).

### Skip 51

Eighteen patients were amenable to skip exon 51. The mean 6MWT was of 400.46 mt at baseline. Mean changes from baseline were of -46.90 mt at 12 months. -86.73 mt at 24 months and -147.34 mt at 36 months. Three were younger than 7 years of age and 15 were above 7 years (Table 3; Figs 1 and 2). Six lost ambulation at 36 months from baseline, their mean age at baseline was 9.23 years (SD ±1.66).

### Skip 53

Twenty-eight patients were amenable to skip exon 53. The mean 6MWT was of 359.46 mt at baseline. Mean changes from baseline were of -28.97 mt at 12 months, -65.15 mt at 24 months and -168.54 mt at 36 months.

Nine were younger than 7 years of age and 19 were above 7 years (Table 3; Figs 1 and 2). Nine lost ambulation at 36 months from baseline, their mean age at baseline was 8.53 years (SD ±2.18).

When we analyzed the 6MWT mean changes from baseline, there was a significant difference between deletions amenable to skip exons 44, 45, 51 or 53 at 24 (*p≤0*.*05*) and 36-months (*p≤0*.*01*) but not at 12 months (*p* = 0.17).

Table 3 reports details of the 6MWT in the study cohort according to mutations amenable to skip exons 44, 45, 51 and 53.

Figs 1 and 2 shows details of the mean changes in the whole study cohort and in the individual subgroups skipping exons 44, 45, 51 and 53.

## Discussion

The need for long term natural history studies reporting the progression of different genotypes over a long period of time has recently been highlighted by the recent Eteplirsen study targeting mutations amenable to skip exon 51 [3]. The study clearly shows that the benefit of treatment is better seen after the first year and becomes more evident with increasing time. These results indicate the need of longer studies to establish the efficacy of drugs with similar mechanism of action. A recent workshop on clinical trial design involving various stakeholders including patients and advocacy groups has however highlighted that patients and their families would not be prepared to enter a study if the duration of the placebo treatment is longer than 12 or 18 months, proposing that natural history data could be used as external controls for longer studies[15].

One of the concerns with using previous natural history studies with patients classified according to their genotype is that the numbers in each subgroup, even in large networks, are relatively small and often include heterogeneous groups in terms of age, severity and other variables. So far, the only available long-term longitudinal prospectively collected data on 6MWT, the measure most commonly used as the primary outcome measure in clinical trials, are limited to the 3-year changes in patients amenable to skip exon 51, using two large national dataset[3]. Less is known about the 3-year changes in other subgroups of boys with mutations such as those amenable to skip exons 44, 45 and 53. This information is relevant because there are current clinical trials specifically targeting these subgroups of mutations and using 6MWT as the primary measure.

In the present study, we report the 36 months longitudinal natural history data in a cohort of patients with deletions amenable to skip exons 44, 45, 51, 53. This was possible as the result of an international effort from tertiary care centers sharing similar standards of care and training.

The 36 month follow up in our study confirms and expands previous findings using the North Star Ambulatory Assessment (NSAA) at 24 months [8] reporting that the difference between subgroups amenable to skip different exons is not significant after 12 months but becomes significant at 24 months. In our study, using the 6MWD, we confirmed that the difference was not significant at 12 months but became significant at both 24 (p≤0.05) and 36 months (p≤0.01).

As anticipated from previous studies [5–8], mutations amenable to skip exon 53 had an overall lower baseline values and more negative changes than the other subgroups, with nearly similar results in the subgroup amenable to skip exon 51. At the other end of the spectrum, the deletions eligible to skip exon 44 had better results both at baseline and on follow up. Deletions amenable to skip for exon 45 were associated with a more variable pattern of changes.

In line with previous studies in larger cohorts of DMD boys including all types of mutations [13], patients below and above 7 years had different patterns of progressions with a difference between the two groups at both 24 and 36 months. In the boys younger than 7 years at baseline there was an overall mean improvement on the 6MWT over 36 months with some relative decline only in the third year. The only exception was the subgroup with patients skipping exon 53 who had an initial mean improvement only in the first year followed by some decline already in the second year and a more obvious decline in the third year with mean values below 300 meters at 36 months. It is of note that the 2 patients with earliest loss of ambulation (age 8 years) were both amenable to skip exon 53.

When we looked at individual mutations, single exon deletions were more often associated with less drastic changes, but there was no consistent or obvious pattern of changes as some individual mutations were often associated with variable functional pattern of progression. These results are consistent with previous observations that single deletions, such as deletion of exon 44 or deletion of exon 45 were each associated with variable phenotypes ranging from Becker or intermediate phenotypes to more typical DMD phenotype with loss of ambulation before the age of 12 years. [16].

In this paper, we were also interested to establish whether the findings observed in a previous paper that a combination of 6MWT and TRF could identify also in the individual subgroups the clinical pattern for a faster decline[14].

In our cohort 13 of the 21 (62%) with a combination of TRF>6 sec and 6MWT <350 m lost ambulation within 36 months, irrespective of the genotype. The main difference was that this combination occurred less frequently and at an older age in the patients amenable to skip exon 44 compared to the other subgroups.

In conclusion, our international effort provides details of the 3 year 6MWT changes in patients with deletions amenable to skip different exons, confirming that with increasing duration of the study the differences between subgroups becomes more obvious. Patients amenable of skipping exon 53 in particular appear to have earlier onset of decline and a higher risk of more drastic changes and loss of ambulation. Patients amenable of skipping 45 had a variable progression.

These findings are compatible with previous observations based on muscle biopsies showing different residual dystrophin expression in these subgroups [16–18]. Other factors such as modifier genes [19–21] that were not studied in our cohort may also account for the variability observed.

Further analysis, taking into account type and regime of steroids in the individual subgroups or individual mutations, was limited by the small numbers and by the variability in age and baseline functional status. In each subgroup there was some variability that could not always be justified by the presence of single deletions, full dose steroids or other variables previously associated with more favorable outcome. Including other variables, such as weight, height and body mass index, that were not systematically noted in our cohort, may also help to define a panel of multiple measures that, as recently shown in other DMD cohorts, may be of help to establish more accurate trajectories of progression using advanced statistical analysis [22, 23].
