# Correction: Long-term natural history data in Duchenne muscular dystrophy ambulant patients with mutations amenable to skip exons 44, 45, 51 and 53

**DOI:** 10.1371/journal.pone.0220714

**Published:** 2019-07-31

**Authors:** Claudia Brogna, Giorgia Coratt, Marika Pane, Valeria Ricotti, Sonia Messina, Adele D’Amico, Claudio Bruno, Gianluca Vita, Angela Berardinelli, Elena Mazzone, Francesca Magri, Federica Ricci, Tiziana Mongini, Roberta Battini, Luca Bello, Elena Pegoraro, Giovanni Baranello, Stefano C. Previtali, Luisa Politano, Giacomo P. Comi, Valeria A. Sansone, Alice Donati, Enrico Bertini, Francesco Muntoni, Nathalie Goemans, Eugenio Mercuri, on behalf on the International DMD group

There is an error in affiliation 9 for author Angela Berardinelli. The correct affiliation is: IRCCS Mondino Foundation, Pavia, Italy.
